# Artificial intelligence hallucinations

**DOI:** 10.1186/s13054-023-04473-y

**Published:** 2023-05-10

**Authors:** Michele Salvagno, Fabio Silvio Taccone, Alberto Giovanni Gerli

**Affiliations:** 1 Department of Intensive Care, Erasme Hospital, Université Libre de Bruxelles, 1070 Brussels, Belgium; 2 Department of Clinical Sciences and Community Health, Università degli Studi di Milano, 20122 Milan, Italy

Dear Editor,

The anecdote about a GPT hallucinating under the influence of LSD is intriguing and amusing, but it also raises significant issues to consider regarding the utilization of this tool. As pointed out by Beutel et al., ChatGPT is a promising large language model (LLM), i.e., a text-based virtual assistant, with knowledge derived from vast training data updated to 2021. It cannot directly access internet data, and, at the moment, it does not have any knowledge after this date. Nonetheless, ChatGPT can retain the information the user provides during a conversation to improve its responses to subsequent questions and inquiries. Indeed, even if it does not have the ability to learn like humans, it can analyze and reprocess what it has learned during the conversation, depending on the complexity of the prompts and the language used. This may account for the divergent response patterns elicited by our prompts compared to those of Beutel et al.

Nevertheless, as stated in the correspondence and previously by Azamfirei et al. [1], the content of the figure in our article, which aimed to compare different studies on a specific intervention, is substantially incorrect. Even though ChatGPT, with which the text was written, does not meet the authorship criteria [2], we are ultimately always responsible for the accuracy of the information provided. Additionally, while using this tool, we have often encountered another type of tricky AI “hallucinations,” which involves apparently correct bibliographic references with known authors and coherent titles, but which are entirely non-existent. Alternative AI tools could be used for this objective, allowing for exploring current scientific databases.

As it should always be done with any step in scientific production and already highlighted in our article, an expert in the field should validate the content generated by ChatGPT, even if it were a simple translation into English from another language. The worldwide enthusiasm and awareness of this tool's benefits and risks bring to mind the Dunning-Kruger effect [3], potentially applicable in the setting of scientific writing, as illustrated in Fig. 1.

**Fig. 1:**
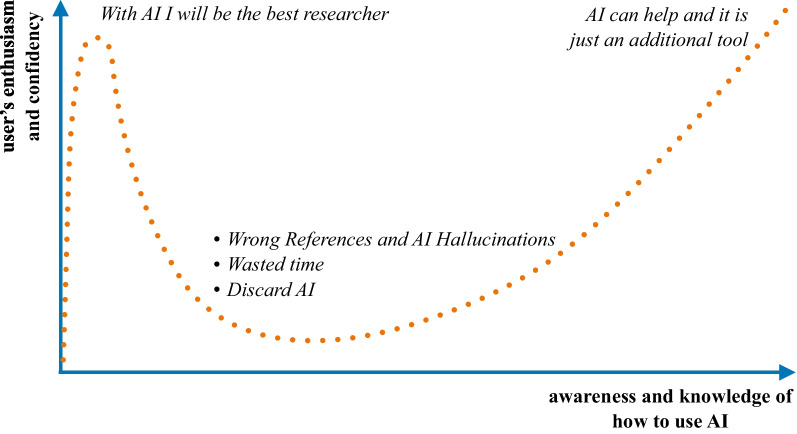
A revised Dunning-Kruger effect may be applied to using ChatGPT and other Artificial Intelligence (AI) in scientific writing. Initially, excessive confidence and enthusiasm for the potential of this tool may lead to the belief that it is possible to produce papers and publish quickly and effortlessly. Over time, as the limits and risks of ChatGPT and other AI are learned, as well as the complexity of their functioning with the need for specific prompts, enthusiasm and confidence decrease. As this awareness increases, ChatGPT and other AIs can become effective and supportive tools in scientific writing, such as computers and internet search engines, finally achieving a conscious and correct usage

Finally, as a colorful detail, we report the case of Kary Mullis, who used LSD and engaged in conversations with an extraterrestrial entity disguised as a raccoon. Still, Mullis' groundbreaking invention of the polymerase chain reaction (PCR) revolutionized medicine, which earned him the Nobel Prize in Chemistry [4]. What would ChatGPT achieve under the influence of LSD? Only time will tell. (However, we do not recommend its consumption).

## Data Availability

Not applicable.

## References

[1] Azamfirei R, Kudchadkar SR, Fackler J (2023). Large language models and the perils of their hallucinations. Crit Care.

[2] Salvagno M, Taccone FS, Gerli AG (2023). Erratum: Correction to: Can artificial intelligence help for scientific writing? (Critical care (London, England) (2023) 27 1 (75)). Crit Care.

[3] Kruger J, Dunning D (1999). Unskilled and unaware of it: How difficulties in recognizing one’s own incompetence lead to inflated self-assessments. J Pers Soc Psychol.

[4] Carlson P. Nobel Chemist Kary Mullis, Making waves as a mind surfer. *The Washington Post*. https://www.washingtonpost.com/archive/lifestyle/1998/11/03/nobel-chemist-kary-mullis-making-waves-as-a-mind-surfer/31e7e720-44e4-49ff-8458-a9822cdcb47e/. Published November 3, 1998.

